# Supplementary figures and images for: Directional and disruptive selection in populations structured by class and continuous ontogeny under incomplete plasticity
Source: PLoS Comput Biol. 2026 Apr 3;22(4):e1013591. doi: 10.1371/journal.pcbi.1013591 (PMC13048448; doi:10.1371/journal.pcbi.1013591)

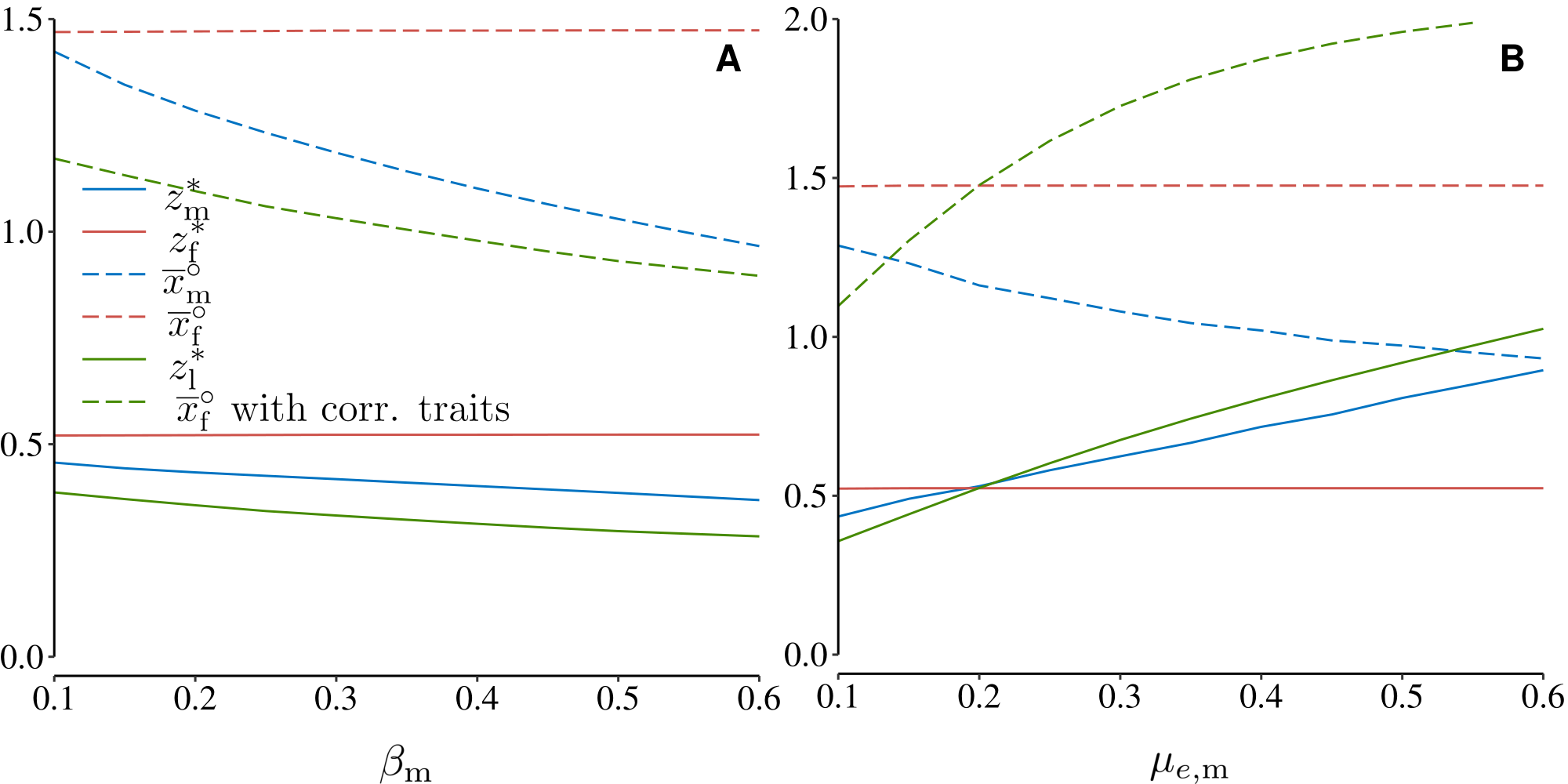

Supplement: S1 Fig — Each panel shows convergence stable growth rates v* (solid lines) and corresponding average sizes (dashed lines) for females (red) and males (blue), as a function of the parameter indicated on the x-axis (computed from eq 36 for trait values and B-99 for average sizes). Trait values under genetic constraint (i.e., identical growth rate in males and females, vl*) and the resulting female size are shown in green (from eq 39). A. Higher cost of growth to survival in males (βm) selects for slower growth and smaller average size in males but does not affect females. B. Higher male extrinsic mortality (μe,m) favours faster growth in males but does not affect females. Under genetic constraints, high values of μe,m also increase the frequency of females, tipping the balance in favour of even faster growth. Parameter values (unless varied on x-axis): c=0.5, α=0.2, μe,f=μe,m=0.1, βf=βm=0.2, κ=1 and K=10000. (TIFF) [file pcbi.1013591.s001.tiff]

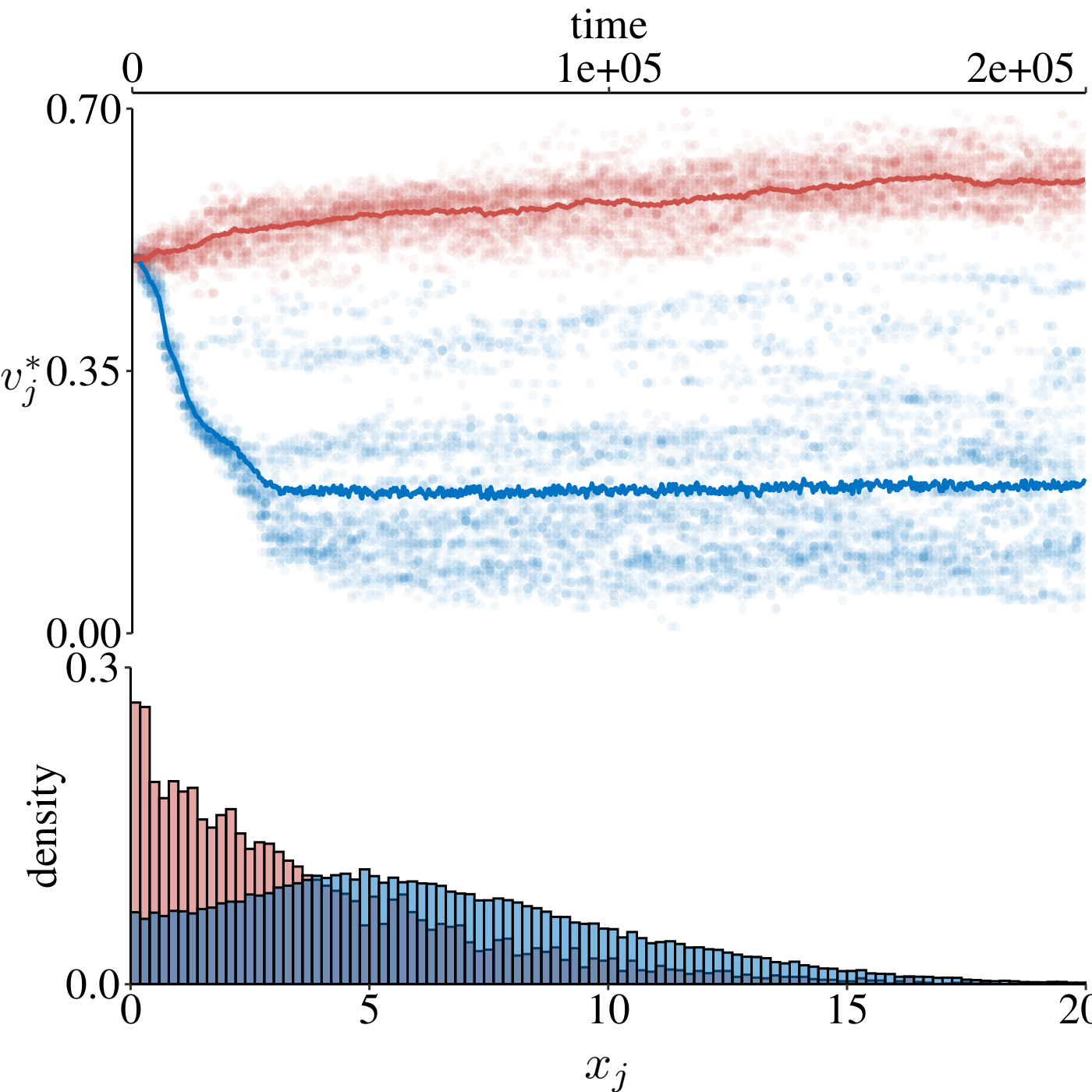

Supplement: S2 Fig — The figure shows results from individual-based simulations (appendix B.3 in S1 Text) with independent growth rates and diploidy. The top part shows the expressed trait values in females (red) and males (blue): randomly sampled allelic values (dots; 20 copies every 10 time units) and population mean (solid lines). The bottom part shows male and female size distributions at equilibrium as histograms (averaged between time steps 105 and 2×10^5^). Parameter values: c=0.5, α=0.02, μe,f=0.1, μe,m=0.01, βf=0.1, βm=0.01, κ=2 and K=10000. (TIFF) [file pcbi.1013591.s002.tiff]

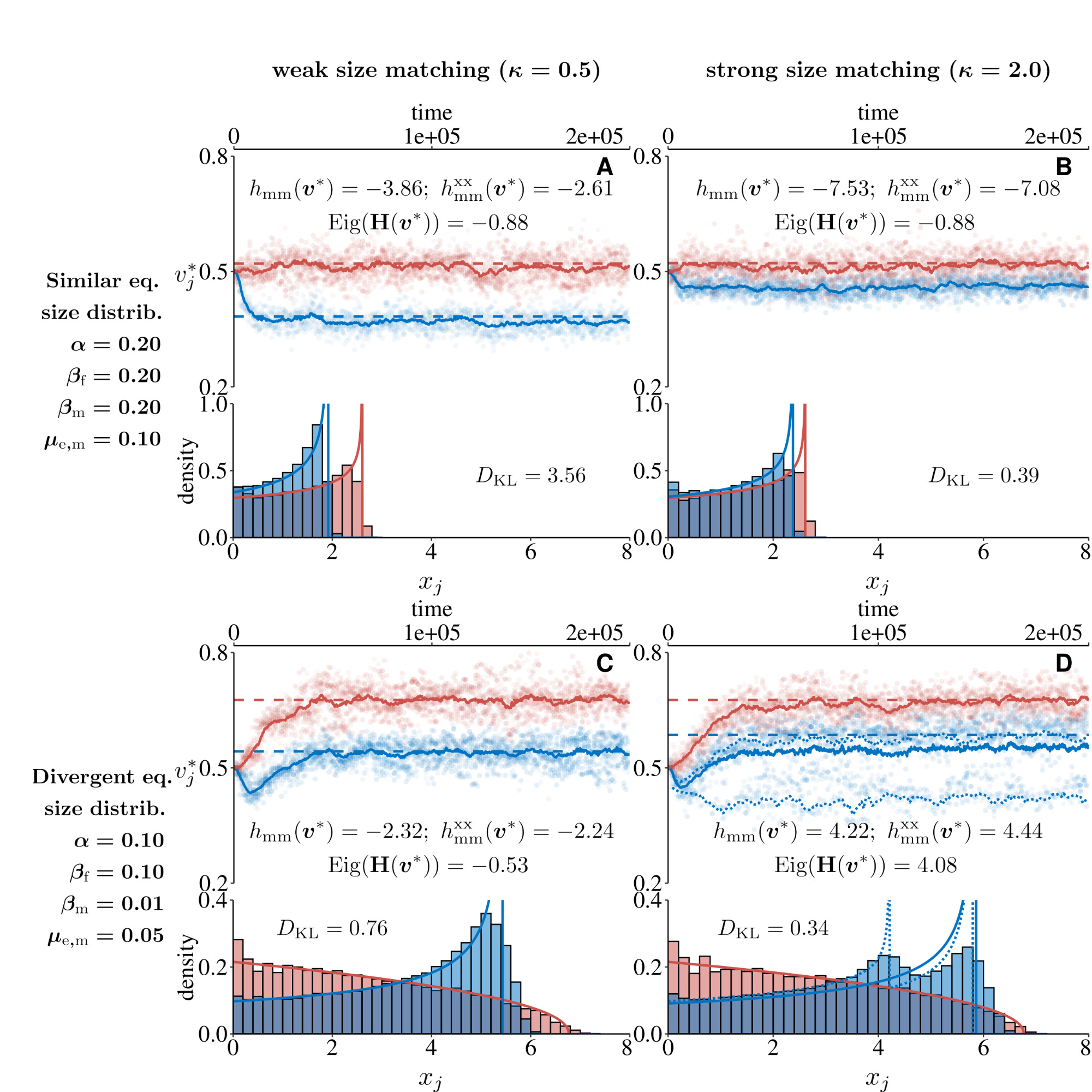

Supplement: S3 Fig — Each panel shows results from individual-based simulations (appendix B.3 in S1 Text). The top part shows the expressed trait values in females (red) and males (blue): randomly sampled allelic values (dots; 10 copies every 10 time units), population mean (solid lines), and analytically predicted convergence stable trait values (dashed lines). Reported values give: the male-specific component hmm(v*) (eq 14); its size-mediated component hmmxx(v*)=0.25∫0∞h^mmxx(a,v*)da (see eqs 37 and A-37, with 0.25 for diploidy); and the leading eigenvalue of H(v*). As expected, when this eigenvalue is negative, the population remains monomorphic (panels A–C). When it is positive, evolutionary branching occurs in males (panel D, split in blue lines). The bottom part of each panel shows male and female size distributions at equilibrium as histograms (averaged between time steps 105 and 2×10^5^), along with the analytically predicted distribution at v* (solid lines, eq 26). The Kullback–Leibler divergence between male and female distributions is also reported. A–B: Male and female distributions are similar in shape as both sexes have the same demographic parameters (see left-hand side of panels for parameter values). However, females grow larger on average due to direct selection on size whereas males only track female size via selection for compatibility. C–D: Sex-specific demography generates asymmetries in size distributions with a higher frequency of small females. When sexual selection is strong enough, this leads to evolutionary branching (panel D) with one morph specialising on mating with smaller females. Under haplodiploidy, net disruptive selection is stronger at the singular trait than in the diploid case, and the smaller male morph evolves smaller genetic values (compare values in Fig 4D and S3D Fig), but the distribution of male size at the evolutionary equilibrium is similar. Other parameters: c=0.5, μe,f=0.1, K=104. (TIFF) [file pcbi.1013591.s003.tiff]

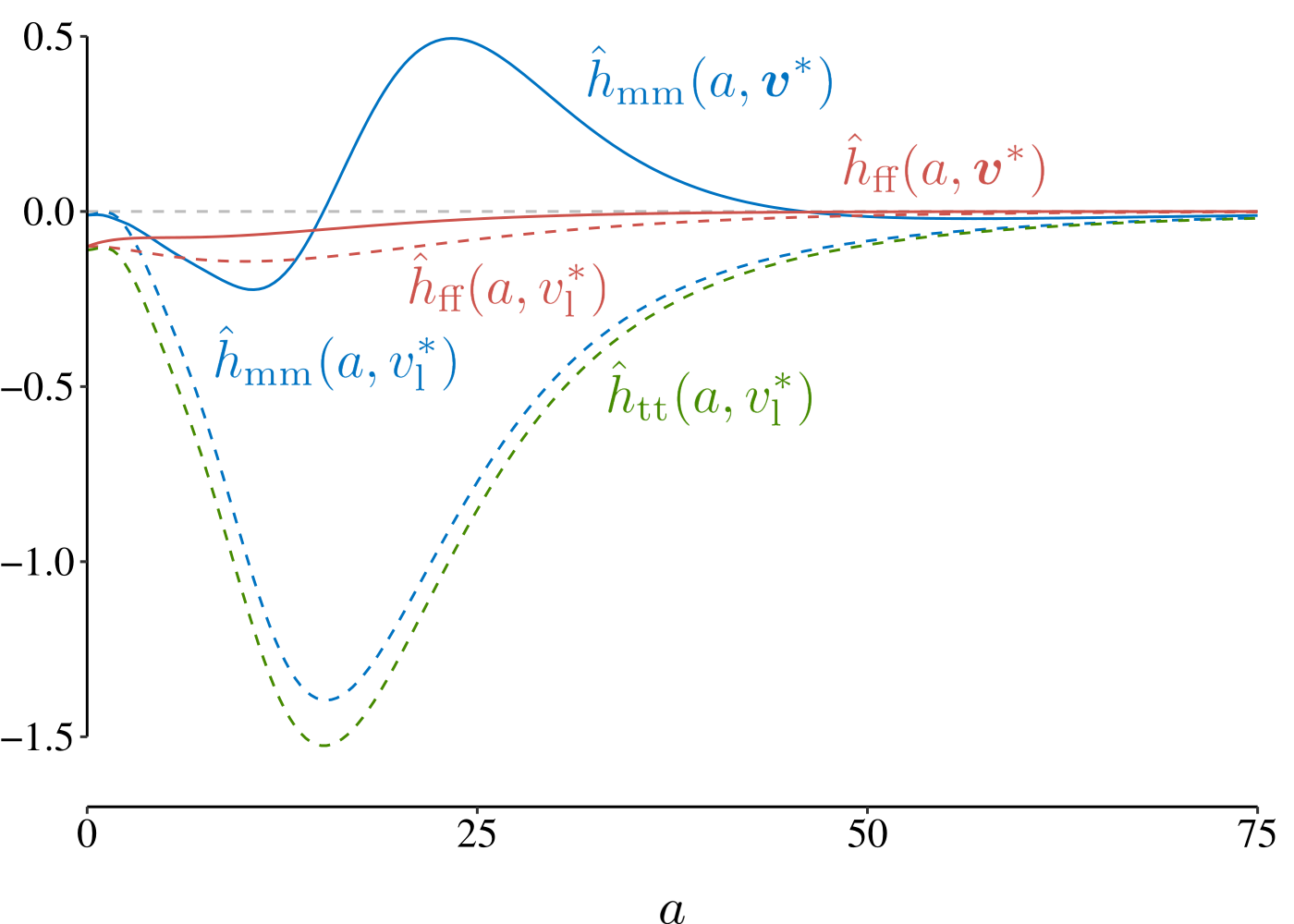

Supplement: S4 Fig — Age-specific quadratic selection on growth (from eqs 35a and 35b) at the convergence stable strategies (v*=(vf*,vm*)=(0.676,0.589) for independent traits; vl*=0.219 for a shared trait), under parameter values that produce disruptive selection on the male growth rate (c=0.5, α=0.1, μe,f=0.1, μe,m=0.05, βf=0.1, βm=0.01, κ=2 and K=104, see Fig 4D). Plain lines correspond to the male and female age-specific components of disruptive selection under independent traits (given by eq 15; recall that here h^jj=h^jj,11). Dashed lines represent the same components, as well as their sum (green line) under genetic constraints (see eq. A-86). (TIFF) [file pcbi.1013591.s004.tiff]

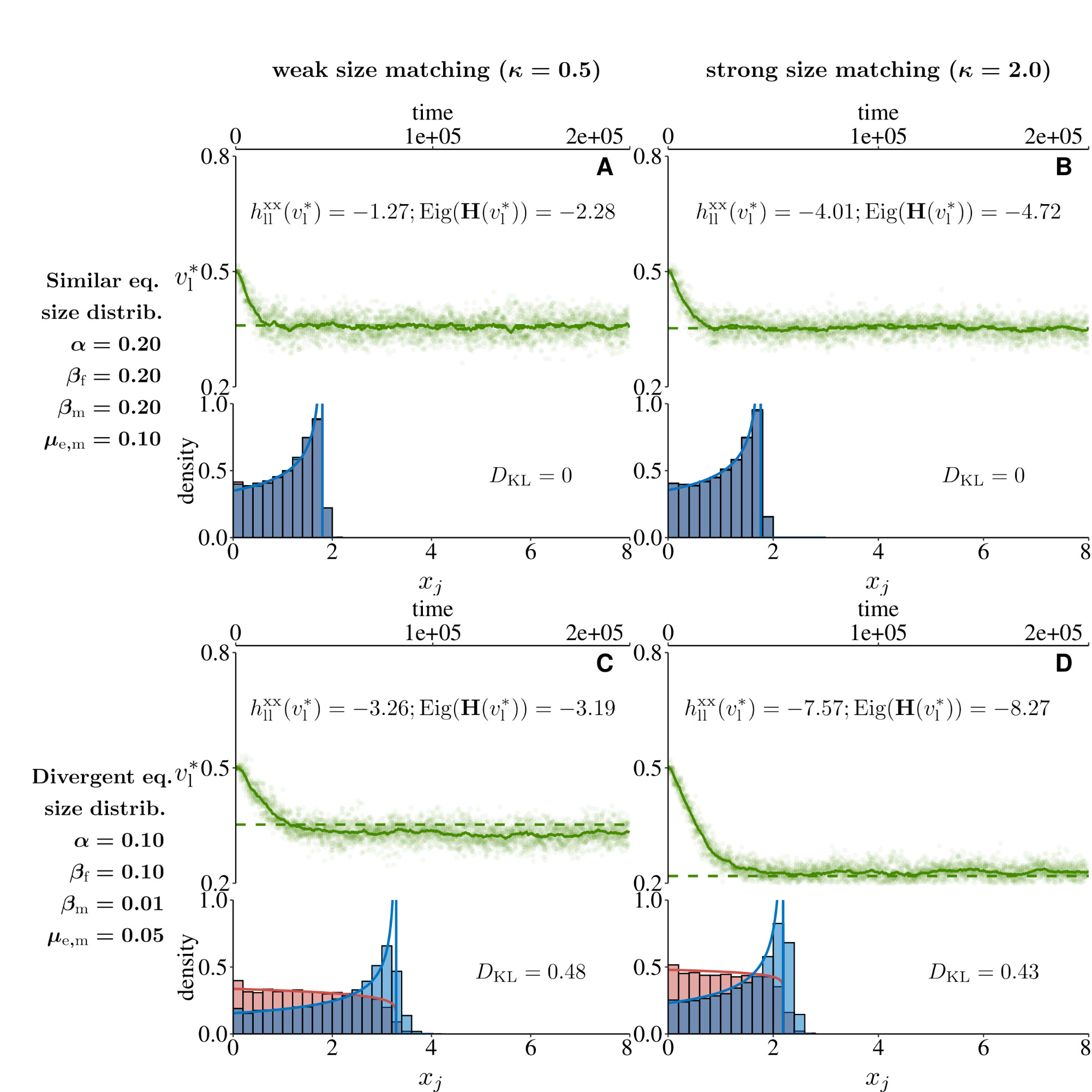

Supplement: S5 Fig — Each panel shows results from individual-based simulations (appendix B.3 in S1 Text). The top part shows the expressed trait values in females and males (green): randomly sampled allelic values (dots; 10 copies every 10 time units), population mean (solid lines), and analytically predicted convergence stable trait values (dashed lines). Reported values give: the size-mediated component of quadratic selection hllxx(vl*)=0.25∫0∞h^llxx(a,v*)da (where h^llxx(a,vl*) is computed as in eq. A-86 using eq. A-37, with 0.25 for diploidy); and the leading eigenvalue of H(vl*). Both are always negative showing size-mediated and net stabilising selection on growth, respectively. The bottom part of each panel shows male and female size distributions at equilibrium as histograms (averaged between time steps 105 and 2×10^5^), along with the analytically predicted distribution at vl* (solid lines, eq 26). The Kullback–Leibler divergence between male and female distributions is also reported. A–B: Male and female distributions are the same since both sexes have the same growth rate and demographic parameters (see left-hand side of panels for parameter values). C–D: Sex-specific demography generates asymmetries in size distributions with a higher frequency of small females. Under genetic constraints, these differences are not sufficient to generate evolutionary branching. Other parameters: c=0.5, μe,f=0.1 and K=10000, and with simulated time steps of 0.2 time units. (TIFF) [file pcbi.1013591.s005.tiff]
