# Supplementary material for: Directional and disruptive selection in populations structured by class and continuous ontogeny under incomplete plasticity
Source: PLoS Comput Biol. 2026 Apr 3;22(4):e1013591. doi: 10.1371/journal.pcbi.1013591 (PMC13048448; doi:10.1371/journal.pcbi.1013591)
Supplement: Attachment — Submitted filename: Review.pdf [file pcbi.1013591.s007.pdf]

Review: of "Directional and disruptive selection in populations structured by class and continuous ontogeny under incomplete plasticity" submitted to *PLOS Computational Biology*

Arthur Weyna, Charles Mullon, Laurent Lehmann

November 27, 2025

This paper presents a general model for studying the evolution of large variety of traits affecting allocation of resources to survival and reproduction, considering structure in class and age, and explicitly considering individual development. The paper presents a framework grounded in the adaptive dynamics framework, with concept and tools such as the CSS or branching, but it is a very large extension of it. This model is so general, yet tractable at least numerically, that it paves the way for addressing long-standing questions, as well as tackling original ones.

The paper is very technical and is based on relatively heavy computations, even though the fundamental elements of the computations are first or second-order derivatives. The model is complicated by structure, not because of the mathematical tools used. The model is complex in structure because it aims at considering at the same time several nested biological layers: genotypes, ploidies, (some) genetic architecture, traits, phenotypes, inter-individual interactions as well as population density. For such a level of integration, I do not see how it would possible to make simpler models, especially if one wants to study how the evolution plays on these different layers.

Despite the complexity of the model and the technicality of the computations, the paper is very-well written. The authors take time to clearly explain as intuitively as possible each part of the equations, with the help of biological justification and background. Even if the content of the paper might not be accessible for most biological readers in details, I think it can be understood by almost anyone with a bit of mathematical background.

By lack of time, I was not able to check computations in details, but I did not find any obvious mistakes or typos.

In short, I think the paper is excellent and is well-suited for a publication in *PLOS Computational Biology.* I have only a few minor comments and suggestions in the following to help further improve the paper.

1 Minor comments

- I am not totally sure it could make sense in the context of the paper, but I wondered if it would be possible to illustrate more precisely the utility of the model by comparing situations with and without considering one of its core aspect. As the main strength of the paper is to integrate in particular age- and class-structure as well as ontogeny, it might be useful to show what does it change to integrate all of it or not. For instance, when studying the example of the evolution of males vs. females traits in section 4, what would be obtained if the ontogeny is neglected?

- L.120: I wondered why the classical denominations 'genotype' and 'phenotype' are not used in the description of the model. It might be useful for some readers familiar with these denominations to clearly define variables and parameters x, z, u, etc. as either phenotype or genotype. In a

similar vein, it might also be useful to specify what are variables or parameters in the model (for instance x are variables, while z are parameters).

- L162: typo "an".
- L167-168: for consistency with "certainty" when $R < 1$, maybe worth it to be more accurate when $R > 1$ and say that there is a positive probability that it invades under the multi-type branching process framework.
- Eq. 1: I think l is not defined close by (even if it's obvious).
- Eq. 3b: Not sure to understand why the decomposition gives $u = v$ in $z = v$ and $u = v$. Please explain.
- I think section 2.3.2 is nice but not necessary and that it can be moved to the appendix in order to shorten the main text.
- After section 3, there is a problem of equations numbering in many places that makes it difficult to follow exactly the computations.
- section 4.1.2: I think this might be worth explicitly mentioning that growth is supposed density independent (I would assume that in general this is not true in nature, rather than on fertility directly).
- Fig. 2B: I did not understand why the plain green and green curves were present at two different values after the extinction threshold? What is the meaning of the two rose curves, the two green curves?
- Fig. 3D: Intuitively I would have predicted that the evolutionary branching would have occurred around the CSS value given by the dashed line for males. This is not the case, and even worse, it seems that one of the two types of males converge to the CSS value while the other do not at all. Why? and in this case what is the meaning of the dashed line for males?
