# Supplementary material for: Directional and disruptive selection in populations structured by class and continuous ontogeny under incomplete plasticity
Source: PLoS Comput Biol. 2026 Apr 3;22(4):e1013591. doi: 10.1371/journal.pcbi.1013591 (PMC13048448; doi:10.1371/journal.pcbi.1013591)
Supplement: Attachment — Submitted filename: review_response.pdf [file pcbi.1013591.s008.pdf]

Response to comments

Review 1:

It was not always clear to me what restrictive assumptions are being made. For example, the set-up seems to exclude environmental effects (including indirect genetic effects) on the phenotype. It also seems to assume that development is deterministic. Some further guidance to the reader would help here.

We agree that some information about our assumptions regarding the genotype-phenotype map were missing from the original submission. To make those assumptions more explicit, we now more clearly state in section 2.1 (l.130-134, 139) that development is deterministic (no stochastic environmental effect on the phenotype), but can depend on deterministic environmental processes. In paragraph 4 of section 2.2 (l.205-214), we now explain in more details how growth and vital rates can depend on environmental variable determined by the resident population. Finally, we have also added a fifth paragraph to section 2.2 (l.215-224), where our assumptions about the genotype-phenotype map are summarized.

More could be done to highlight the new insights from the example at the end, especially in the abstract.

We have now added extra information related to the results obtained with our example model in paragraph 3 (l.890-892) and 5 (l.921-923) of the discussion. We also added more qualitative information about these results in both the abstract (l.12-13) and the author summary (l.28-31).

Can evolutionary branching ever lead to the maintenance of more than two alleles in the example? Naively I would think that if selection for assortative mating is very strong, males should split up into even more morphs.

Branching can indeed lead to the maintenance of more than two alleles in our example model. In agreement with the reviewer's intuition, this requires strong sexual selection, but also that the equilibrium distribution of female size is wide enough that many different non-overlapping male morphs can coexist. This is akin to results from niche partitioning model where the breadth of the resource distribution modulates the number of morphs that coexist in the long-term (e.g., Schmid et al., 2023). To illustrate this result, we have now added a supplementary figure (fig. S2 in the revised manuscript), which is a simplified version of fig. 4D (ex fig. 3D), but where mortality and growth parameters have been modified to allow for a wider size distribution (in particular setting alpha=0.02). This displays the coexistence of many male morphs, and is referred to on line 784-785.

There is a substantial literature on size-assortative mating in various species of mollusc. Perhaps the authors could link their example to this literature.

We agree that links to the empirical literature are needed to illustrate the applicability of our example model. After some literature review however, we found that size-assortative mating in mollusks is often attributed to a preference for larger-sized partners (irrespective of the size of the chooser), occurs in hermaphrodites or is studied in the context of pre-existing polymorphisms/divergence due to environmental heterogeneity (as in Littorina sea snails). Thus, to better link our example to the literature while trying to stay as close as possible to our own assumptions we reference (in the last paragraph of section 4.1.3, l.689-694) three studies where data suggests that it is size matching within a breeding pair that determines individual fitness (two in cichlids and one in crickets).

L44-60: Another point is that if plastic traits are governed by a large number of loci, each of which is only expressed in particular circumstances, then selection on each locus might be very weak.

We agree that we should then expect a reduction in the strength of selection on each age-specific locus, as reaction norms become more fine-grained. We thank the reviewer for bringing up this point. We have now added a sentence in the second paragraph of the introduction (l.57-58) that we hope reflects this argument.

L61, 88: 'traits'

This has been corrected.

L116: It might be helpful to specify that n_x is independent of age.

We agree that the original submission did not clearly state that n_x is independent of age. We now do so in the first paragraph of section 2.1 (l.123-124).

Eq. 1: The survival probability function used in this equation is not introduced until two paragraphs later. Some signposting or rearrangement would be helpful.

The definition of survivorship did come up too late after eq. (1) in the original submission (two paragraphs later). We have now reorganized section 2.2 (l.192-224) so that all introduced quantities are described within a few lines of their first mention.

Table 2: The notation v for the singular strategy hasn't been introduced yet. Maybe it could be added to the table legend.*

We have followed the reviewer's advice and added to the caption of table 2 a reference to eq. (5) and (21),

L222: Does this actually correspond to any mathematical definition of curvature? I know that similar claims are made frequently in the theoretical literature on quadratic selection and certainly the second derivative is related to curvature. But most definitions of curvature have normalisation terms that depend on the first derivative and I see no guarantee that such terms will be negligible here.

We agree that curvature has a very specific definition (that includes normalization), which does not fit with our use of the term. Even though similar phrasing is common in the field, we agree that this could lead to unnecessary confusion. To solve that issue, we have now re-written the last paragraph of section 2.3.1 (l.250-256), removing that term and better describing what aspects of the adaptive landscape are measured by Hessian matrices.

L236-7: I don't understand why mutation can't move traits along a boundary.

Mutation can indeed move traits along a boundary. We agree that our initial wording made it seem like this was neglected. We have now rewritten this part (l.266-270) to increase clarity.

L240: Mutational effects being exactly equal across traits is only one possible type of constraint on the mutational effects distribution, but it seems as if you treat this as the only form of such a constraint. See also L277 and L563 onwards.

We agree with the reviewer that mutational effects being fully correlated across traits is not the only possible type of constraints on the distribution of mutation effects. Under our assumptions however, it is the only type of such constraints that can lead to a situation where eq. (4) is true (i.e., gradual evolution has reached a singular point) without eq. (5) being true (i.e., directional selection on each trait has vanished). In other

words, even with strong mutational covariance, mutations can always be drawn that change one trait without affecting the other. Thus, on the long-term and under rare mutations, traits can be seen as evolving independently as long as mutational effects are not fully correlated across traits. We have now modified the second paragraph of section 2.3.2 (l.272-278) and the last paragraph of section 3 (l.596-604) to make it more explicit that we are not assuming that other types of constraints are absent.

Box 1: The co-states have no yet been defined. Please signpost.

Boxes are required to appear right after their first mention in the main text according to the formatting guidelines. In the original submission, box 1 appeared early because of a reference to it in the first paragraph of section 3.1.1. To solve this issue, we have removed this early (and non-necessary) reference from the revised version. Box 1 now appears after the introduction of costate variables (p.17).

P15, P26: The equation numbering does not continue correctly after the two boxes.

We unfortunately missed this problem when proof reading. We apologize for this slip-up and thank both reviewers for carrying on with the review despite the incurred discomfort. This has now been corrected.

L630: Please specify that it is the per-male rate of egg production.

The quantity $(1 - \tilde{c}^{\circ})N_T^{\circ}\chi^{\circ}_f(x_f)\, f_f(x_f, v)$ is not, in fact, the per male rate of egg production. The quantity $(1 - \tilde{c}^{\circ})N_T^{\circ}\chi^{\circ}_f(x_f)$ is the absolute density of females of size x_f, while $f_f(x_f, v)$ is the fecundity of one such female, so that the product of these two quantities is the absolute rate of egg production by all females of size x_f collectively. We have now rewritten this sentence (l.675-678) to remove ambiguity.

Fig. 3: This figure is quite hard to read, requiring switching back and forth between the legend and the figure. For example, finding the full set of parameter values for each panel requires reading both the top and side labels in the figure and the remaining parameters in the legend. Some redundancy and better signposting would be very helpful here.

As an effort to make this figure (now fig. 4 in the revised version) more easily readable, we have first added titles to its two rows (columns), which indicate the biological meaning of the parameter values used in each row (column). We also believe that, in the original submission, much of the difficulty in reading this figure came from the fact that the reader was not introduced to the equilibrium distribution of size (its shape and dependence on parameter values), such that the bottom parts of each panel of the figure were not immediately understandable. We have now added a figure (fig. 1 in the revised version) displaying eq. (26), and showing how the predicted shape of the resident equilibrium size distribution varies with parameters (and corresponding to plain lines in the bottom parts of each panel in fig. 4). We hope that this visual aid will make fig. 4 easier to interpret.

Eq. 21: Please remove the comma at the end of the equation (grammatically the equation functions as the subject of the following sentence – the comma suggests instead that the equation functions as a sentence in its own right).

Corrected.

L707-8: Missing close parenthesis.

Corrected.

Review 2

I am not totally sure it could make sense in the context of the paper, but I wondered if it would be possible to illustrate more precisely the utility of the model by comparing situations with and without considering one of its core aspect. As the main strength of the paper is to integrate in particular age- and class-structure as well as ontogeny, it might be useful to show what does it change to integrate all of it or not. For instance, when studying the example of the evolution of males vs. females traits in section 4, what would be obtained if the ontogeny is neglected?

Appendix B.1.4 of the original submission actually contained results giving expressions for the singular growth rate of the example section in the absence of class-structure and ontogeny. As made explicit by the reviewer's concern, however, we did not reference these results in the main text. We now mention this appendix in section 4.1.1 (l.627-628). We also added a paragraph at the end of section 4.2.3 (l.786-803), where we explain in more details what new features ontogeny brings compared to previous compatibility-based sexual selection models. Specifically, ontogeny in our model is responsible for variability in the states of mating females, triggering diversification and specialization. Other models that demonstrated diversification in mating strategies relied instead on prior female genetic diversification.

L.120: I wondered why the classical denominations 'genotype' and 'phenotype' are not used in the description of the model. It might be useful for some readers familiar with these denominations to clearly define variables and parameters x, z, u, etc. as either phenotype or genotype. In a 1similar vein, it might also be useful to specify what are variables or parameters in the model (for instance x are variables, while z are parameters).

We agree that making a link between the quantity used here and the fundamental concepts of genotype and phenotype is required for maximum clarity. We have now added a third paragraph to section 2 (l.215-224), describing how traits, states and vital rates (in our terms) can all be understood as phenotypes, i.e. functions of the genotype (the set of alleles carried by an individual) and the environment.

L162: typo "an".

This has been corrected.

L167-168: for consistency with "certainty" when $R < 1$, maybe worth it to be more accurate when $R > 1$ and say that there is a positive probability that it invades under the multi-type branching process framework.

We agree that the original submission was going too fast on that point and thank reviewer 2 for requesting clarification. We have now modified this part (l.181) to reflect the reviewer's point that there is only a positive probability that a mutant invades when its R > 1.

Eq. 1: I think l is not defined close by (even if it's obvious).

The definition of survivorship did come up too late after eq. (1) in the original submission (two paragraphs later). We have now reorganized section 2.2 (l.192-214) so that all introduced quantities are described within a few lines of their initial mention.

Eq. 3b: Not sure to understand why the decomposition gives $u = v$ in $z = v$ and $u = v$. Please explain.

In eq. (3b), we use the chain rule to decompose derivative of *∂R0(u, v)/∂u* (evaluated at the resident *u*=*v*) into the product of two derivatives. The first is the effect *∂R0(u, v)/∂z* of the trait *z* on *R0* (recall that *R0* is a function of *z*, which we keep implicit here) evaluated at the resident trait (*z*=*v* since *v* is the resident trait, see the first paragraph of section 2.2*)*. The second is the effect *∂z(u, v)/∂u* of the genetic value on the trait, evaluated at the resident genetic value *v.*

We believe the confusion here comes both from the implicit dependence of *R0* on the trait *z*, and from the fact that that the traits and genetic values of a resident individual are equal under our assumption. We now recall the two aspects below eq. 3C (l.236).

I think section 2.3.2 is nice but not necessary and that it can be moved to the appendix in order to shorten the main text.

Section 2.3.2 describes some of the fundamental concept used in the paper and how long-term evolution proceeds. After contemplating moving into an appendix, we concluded that this would be make following the argument of the paper unnecessarily hazy. Even though we do not produce new results in this section 2.3.2, we believe that the synthesis and explanations it contains have real value for understanding and motivating the results that follow. We thus chose to keep this section in the revised version.

After section 3, there is a problem of equations numbering in many places that makes it difficult to follow exactly the computations.

The same issue was brought forward by Reviewer 1. We unfortunately missed this problem when proof reading. We apologize for this again and thank both reviewers for carrying on with the review despite the incurred discomfort. This problem has now been corrected.

section 4.1.2: I think this might be worth explicitly mentioning that growth is supposed density independent (I would assume that in general this is not true in nature, rather than on fertility directly).

We agree that a more realistic model would include more explicit resource budgeting, including the effect of the resident population on resource acquisition and allocation. We have now added a sentence before eq. (22) (l.636-637) to make our assumption that growth is independent of the environment more explicit.

Fig. 2B: I did not understand why the plain green and green curves were present at two different values after the extinction threshold? What is the meaning of the two rose curves, the two green curves?

The rose and green curves report the CSS trait values and average size in females, in the case of independent and correlated traits respectively. The reason why values are reported even beyond the extinction threshold is that the model mathematically holds even for negative values of the total population density N_T°. This being said, we agree with the reviewer that these values make little to no biological sense, and that reporting them without careful explanation brings confusion. We have now modified the figure so that curves are shaded past the extinction threshold, and added explanations in the caption of fig 3 (ex fig2, p.35).

Fig. 3D: Intuitively I would have predicted that the evolutionary branching would have occurred around the CSS value given by the dashed line for males. This is not the case, and even worse, it seems that one of the two types of males converge to the CSS value while the other do not at all. Why? and in this case what is the meaning of the dashed line for males?

Under gradual evolution (very small genetic variance), we agree with the reviewer that we should expect disruptive selection to play a role (and trigger branching) only when the population has reached the singular trait and direction selection has vanished. In the simulation used to produce fig4D (ex. fig. 3D) however, we

do have some genetic variance, and parameter values have been chosen to produce strong disruptive selection. This results in disruptive selection playing a role before directional selection has completely vanished (before the population has converged to the CSS). We have now added a sentence explaining this in the second paragraph of section 4.2.3 (l.781-783).

Regarding the asymptotic position of each male branch relative to the CSS, we see no reason to believe that one branch should or should not end up close to that value. Here, the position of each branch is influenced in complex ways by ploidy (see fig. S3 where the positions are different), branch frequencies, and by the extreme asymmetry in the distribution of “resources” (i.e., female size).
